# Simulating targeted vaccination strategies with network-based and agent-based models: A scoping review

**DOI:** 10.1371/journal.pgph.0005584

**Published:** 2026-06-10

**Authors:** Amera Al-Amery, Rabiah Al-Qudah, Jose Herrera-Diestra, Melissa Zeynep Ertem

**Affiliations:** 1 Department of Computer Information Systems, Jordan University of Science and Technology, Irbid, Jordan; 2 Department of Computer Science and IT, Abu Dhabi University, Abu Dhabi, United Arab Emirates; 3 Department of Mathematics, Tarleton State University, Stephenville, Texas, United States of America; 4 School of Systems Science and Industrial Engineering, State University of New York at Binghamton, Vestal, New York, United States of America; PLOS: Public Library of Science, UNITED STATES OF AMERICA

## Abstract

Understanding the structural characteristics of contact networks in network-based and agent-based models is essential for evaluating and optimizing targeted vaccination strategies in infectious disease control. In this study, a comprehensive scoping review was conducted to explore simulation-based research that examines the interaction between network structures and targeted vaccination interventions during epidemics. The review highlights the importance of understanding how different network structures can influence the effectiveness of vaccination strategies in controlling disease outbreaks. A total of 39 studies published between 2018 and 2025 from different databases such as Scopus and Web of Science were systematically analyzed. The review illustrates the current gap in modeling practices and identifies the need for more realistic network representation and evaluation of a hybrid intervention as a future research priority. The time window (2018–2025) was chosen to capture the most recent generation of simulation-based studies that have been accelerated during and after the COVID-19 pandemic. This review reflects the emerging nature of the intersection between network science and epidemic modeling. Providing well-structured insights to support the formulation of effective vaccination strategies.

## Introduction

Healthcare systems encounter significant challenges in detecting and controlling the spread of infectious diseases, as well as in preventing the overburdening of healthcare resources. Both pharmaceutical interventions (such as vaccination) and non-pharmaceutical measures (such as quarantine and social distancing) are designed to delay the outbreak of the pandemic, to assist healthcare systems with preparations for outbreaks, to reduce the spread of disease, and to assist researchers in finding a vaccine or drug if one has not yet been developed [1]. A targeted vaccination is an essential strategy to stop the spread of the disease [2].

Contact networks are one of the most effective tools used in understanding how diseases spread through populations [3]. In these networks, individuals are represented by nodes, and the interactions—such as physical proximity, communication, or social behavior are represented by edges between individuals. A network can be more complex; it may contain multiple types of nodes or edges, and nodes or edges may have a variety of properties, numerical or otherwise. When considering a social network, for example, the nodes may be men or women, individuals with different characteristics. The structure of the networks is not random; it reflects real-world patterns such as clustering, hubs, and community structures, which significantly impact epidemic dynamics by affecting outbreak size, duration, and the effectiveness of control strategies [4,5].

A highly clustered structure, or a high tendency for individuals’ contacts to be interconnected, allows an epidemic to spread rapidly within a cluster once it has entered, making epidemics more localized within communities [6]. Hubs or the individuals with a significantly higher number of connections can act as highly efficient spreaders due to their extensive reach. Identifying these hubs is crucial for controlling epidemics, as immunizing them can be an effective strategy to reduce disease transmission [7].

Meanwhile, a strong community structure characterized by tightly interconnected groups within a network with limited connections between them can result in long-lasting epidemics with high transmission generation but potential low overall incidence. Conversely, less structured networks may be susceptible to more explosive outbreaks [8].

Unlike classical compartment models that assume homogeneous mixing within populations, which is reasonable when contact patterns are random and homogeneous, heterogeneity is more common in real-world populations. Network representations are explicitly designed to capture heterogeneity, clustering, and connectivity in contact patterns, all of which have a critical impact on epidemic dynamics and intervention efficacy [9].

Many theoretical epidemiological studies have begun to utilize contact networks as an effective way of modeling populations, and to simulate reality in epidemiological modeling [10–13]. An important consideration when establishing the network model with the contact network is the type of network to use, as the network structure differs across network types [14]. Network structure has a major impact on the dynamics of the epidemic [15]. When there is no immune strategy, the network structure is the key factor determining the size of the disease outbreak under the law of long-term evolution.

**Fig 1:**
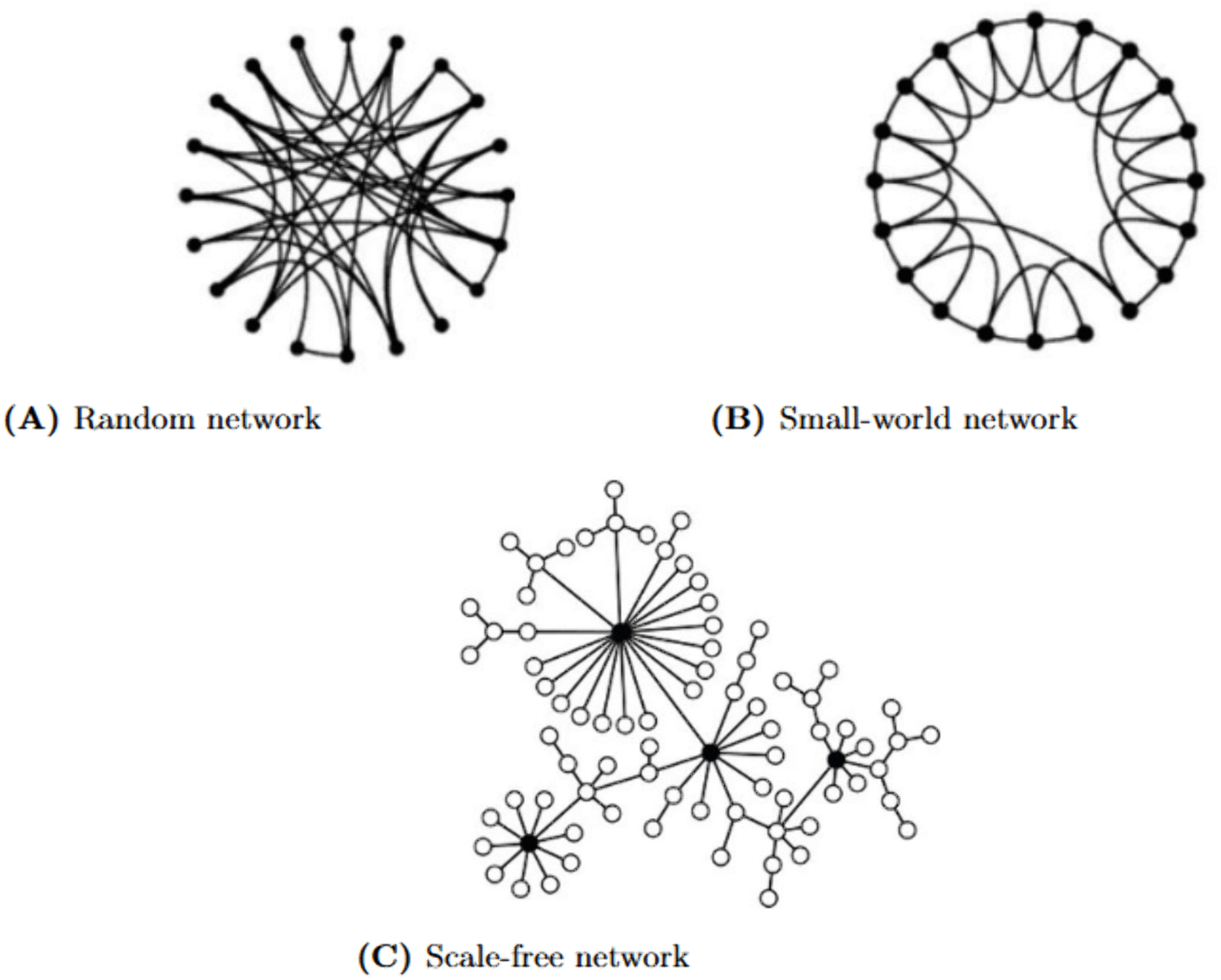
Topology structure for the common networks used in epidemiological modeling. **(A)** Random networks assume that contacts among individuals are uniformly distributed. **(B)** Small-world networks exhibit high clustering and short path lengths. **(C)** Scale-free networks contain a small number of highly connected hub nodes and a large number of weakly connected nodes [18].

In order to model the spread of the disease, a set of contact networks is used: a scale-free network (e.g., Barabási–Albert), which is characterized by highly heterogeneous degree distributions, with a few highly connected nodes (hubs) dominating connectivity [16]. In disease propagation, these nodes control the transmission, leading to rapid spread and allowing diseases to persist even at low infection rates [14]. Small-world network (e.g., Watts–Strogatz) has high clustering (dense local connections) and short path lengths (global connectivity), reflecting the structure of many real-world complex systems and makes it very appropriate for studying the spread of epidemics [17]. Moore and Newman [13] studied some simple models of disease transmission on small-world networks, while Small and Tse [12] applied the small-world model to simulate the spread of the SARS virus in Hong Kong. Random networks (e.g., Erdős–Rényi), which assume that contacts are formed uniformly at random, are often used as a baseline model. The topology structures of the most commonly used networks in epidemiology are illustrated in Fig 1A–1C.

The use of contact networks in these studies typically involves three steps: First, construct a realistic network model of the contact patterns. Second, individual-based versions of compartmental epidemic frameworks, such as SIR (Susceptible–Infectious –Recovered) and SEIR (Susceptible–Exposed–Infectious–Recovered) models— which divide the population into compartments representing different disease states—are used to evaluate how infections spread through populations based on the underlying characteristics of the disease and the structural characteristics of the network [13,19]. The third step involves manipulating the network to model epidemic control strategies such as targeted quarantine or vaccination, guided by the network’s structure (e.g., node degree) [20]. Within this framework, population interventions can be simulated by modifying the response of individuals facing potential infection, by changing node and edge attributes relevant to the transmission of the disease: for example, removing nodes to simulate vaccination, removing edges to represent contact reduction, or reducing edge weights to model reduced transmission probability [21].

Vaccination strategies that involve modeling-based prioritization rules, which identify the order in which individuals or groups are vaccinated based on network position, demographic attributes, behavior, or contact patterns, are a primary focus of this research across various studies. Baseline approaches involve random immunization, which, while simple, often require high coverage to be effective [22].

However, more advanced and informed strategies are needed, especially when the vaccine doses are limited. These strategies can be categorized as follows: (i) topology-based strategies, which prioritize individuals based on network metrics such degree, which represents the number of edges (connections) to a node in the network; betweenness, which reflects a node’s role as a bridge connecting different parts of the network; and eigenvector centrality, which measures a node’s importance based on the number of connections to other highly connected nodes [23]; (ii) demographic-based strategies, which target individuals based on attributes such as age, occupation, or health condition [24]; (iii) behavioral or activity-based strategies, which target individuals with high contact frequency or risk-related behaviors [25]; and (iv) reactive (contact-based) strategies, such as ring vaccination, which is triggered by contact tracing or case detection [26].

This scoping review includes studies that use contact networks to study the impact of targeted vaccination strategies. These strategies were considered in our study because they are most commonly used and evaluated in network-based and agent-based simulation studies, which allow us to conduct a consistent comparison among models. Although many studies have been conducted to model the dynamics of infectious diseases, focusing on compartmental modeling, the systematic synthesis of network-based studies on targeted vaccination strategies remains limited. Our review narrows the scope to network-based and agent-based simulations. Specifically, we identify the types of network models used and explore how the targeted vaccination strategies are developed and assessed within these frameworks.

## Materials and methods

**Fig 2:**
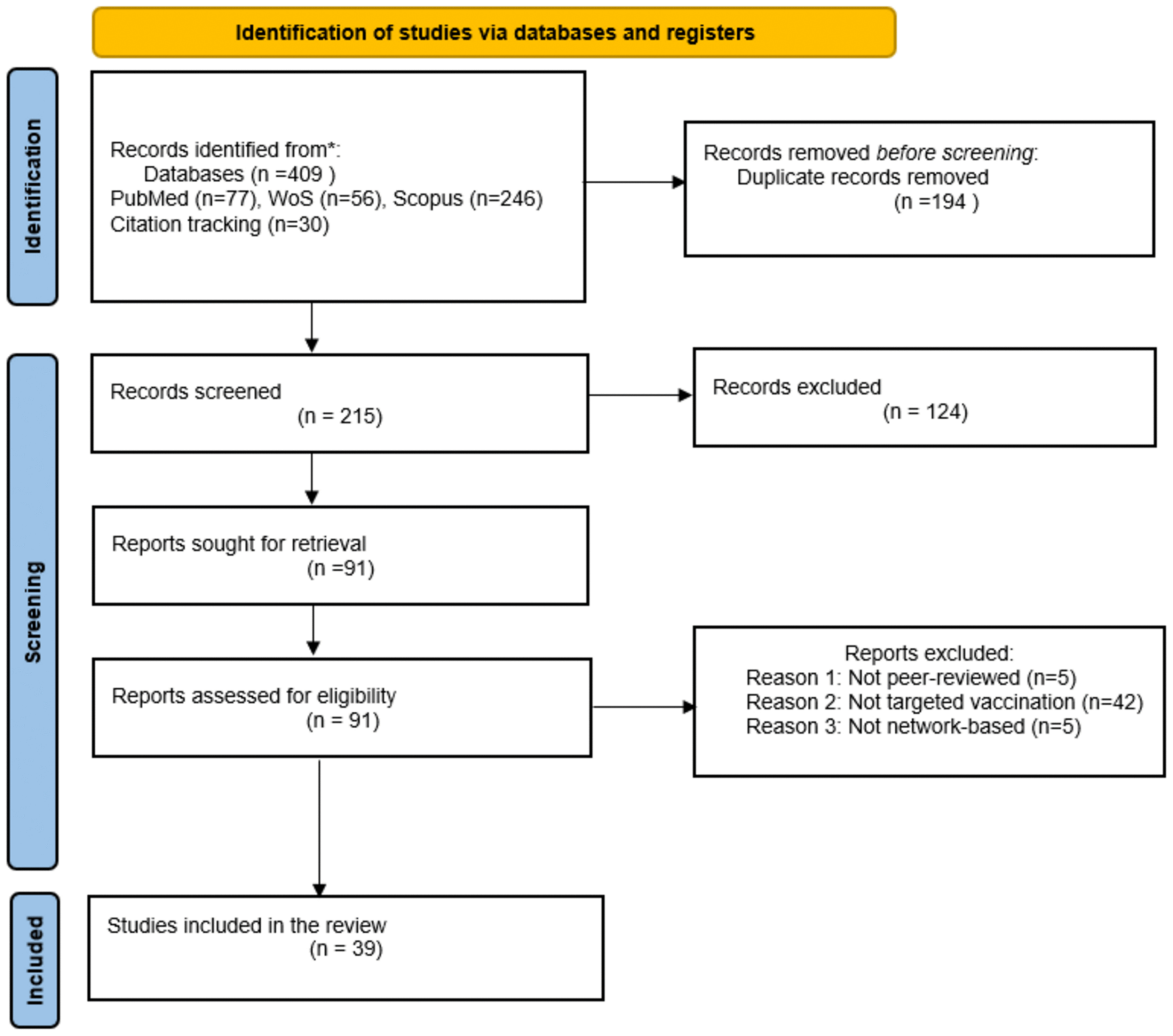
PRISMA flow diagram showing the screening and selection process of included studies.

This scoping review was conducted in accordance with the PRISMA-ScR (Preferred Reporting Items for Systematic Reviews and Meta-Analyses Extension for Scoping Reviews) guidelines [27]. All stages followed the PRISMA-ScR checklist to ensure methodological transparency (S1 Checklist). The screening and selection process is illustrated in Fig 2.

### Identifying the research question

To effectively achieve our objectives, we have established three research questions, accompanied by their justifications, as outlined in Table 1.

**Table 1:** Reserach questions and justification.

Research Question	Justification
**How did the studies utilize contact networks in targeted vaccination interventions in terms of nature, dynamics, and structure?**	The aim of this question is to reveal the types of networks in terms of origin (empirical, theoretical, hybrid), dynamics (static vs. dynamic), and structural type (e.g., scale-free, small-world) used to model the targeted vaccination. The structure of the network strongly affects the transmission and the impact of the intervention [1,28,29], so answering this question can highlight trends and methodological preferences in the literature.
**What vaccination strategies are implemented in the models?**	The objective of this question is to identify the vaccination strategies. It shows the gaps in current approaches and helps researchers explore more effective vaccination strategies.
**What outcomes are used to evaluate these strategies, and how effective are they in controlling disease spread (e.g., infection reduction, delay, etc)**	Vaccination strategies have two core objectives: to delay the peak size of infections and to reduce the final infected population [30]. Analyzing these outcomes helps determine how well different strategies perform in practice and provides a basis for comparing the effectiveness of different approaches.

### Identifying relevant studies

After establishing the research questions, we identified the core concepts relevant to the scope of this review. A comprehensive list of keywords, including synonyms and closely related expressions, was used to maximize search sensitivity across databases. These keywords were combined using the OR operator and the AND operator, resulting in a complete search string that accurately captures studies at the intersection of all identified concepts. The main concepts and their related keywords are illustrated in Table 2.

**Table 2:** Key concepts and keywords for the search strategy.

Concept	Example Keywords and Synonyms
**Contact Networks**	“contact network,” “social network,” “community networks,” “network-based model,” “epidemic network,” “agent-based model”
**Vaccination Strategies**	“targeted vaccination,” “vaccination,” “vaccination strategies,” “immunization,” “immunization strategy,” “vaccination intervention,” and “immunization intervention”
**Infectious Disease Control**	“infectious disease control,” “epidemic control,” “disease mitigation,” “containment strategy,” “transmission control,” “disease prevention,” and “public health intervention”

To capture relevant studies, we conducted searches across several major databases, including Scopus, PubMed, and Web of Science, using the same set of queries, see Table 3. Moreover, we applied snowball sampling by reviewing the reference lists of relevant review articles identified during our screening process. A list of all identified records was imported into Rayyan, an open-access software program. In addition to removing duplicate records, the title and abstract of the remaining records were screened for eligibility. We utilized Rayyan’s AI screening and highlighting features, but screened each record manually. Each full-text article was carefully reviewed by a team of at least two researchers, ensuring a thorough and independent examination of the material.

**Table 3:** Scopus search terms.

Query number	Query
Scopus (Q1)	TITLE-ABS-KEY((“social network” OR “contact network”) AND (“vaccination” OR “targeted vaccination” OR “vaccine strategy”)) AND ABS((“mathematical model” OR “compartmental model” OR “SIR model” OR “SEIR model” OR “epidemiological model”)) AND PUBYEAR > 2009 AND PUBYEAR < 2026 AND (LIMIT-TO (DOCTYPE,“ar”)) AND (LIMIT-TO (LANGUAGE,“English”))
Scopus (Q2)	TITLE-ABS-KEY((“social network” OR “contact network”) AND (“vaccination”)) AND ABS((“mathematical model” OR “compartmental model” OR “SIR model” OR “SEIR model” OR “epidemiological model”)) AND (LIMIT-TO (DOCTYPE,“ar”)) AND (LIMIT-TO (LANGUAGE,“English”))
Scopus (Q3)	TITLE-ABS-KEY((“agent-based model” OR “individual-based model”) AND (“vaccination”) AND (“disease transmission”)) AND (LIMIT-TO (DOCTYPE,“ar”)) AND (LIMIT-TO (LANGUAGE,“English”))
Scopus (Q4)	TITLE-ABS-KEY((“agent-based model” OR “network model” OR “contact network” OR “social network”) AND (“vaccination” OR “vaccine strategy” OR “targeted vaccination”) AND (“disease transmission” OR “epidemic control” OR “infectious disease spread”)) AND ABS((“mathematical model” OR “compartmental model” OR “SIR model” OR “SEIR”)) AND (LIMIT-TO (DOCTYPE,“ar”)) AND (LIMIT-TO (LANGUAGE,“English”))

### Eligibility criteria

We included peer-reviewed simulation-based and modeling-based studies published in English between 2018 and 2025. The 2018–2025 window was selected to capture the most recent and methodologically comparable generation of simulation-based studies, particularly following COVID-19, see Table 4 for the eligibility criteria.

**Table 4:** Eligibility criteria and their rationale.

Eligibility criteria	Rationale
**Inclusion criteria**	
Peer reviewed	Included to ensure that studies are scientifically credible and have been reviewed by peer experts in the field
Simulation-based or modeling studies	Included to ensure that the review focuses on studies that provide structured insights into the effectiveness of targeted vaccination strategies in network-based epidemics models
Use of network-based models	Included to ensure alignment with the review objective of assessing the vaccination strategies in the context of contact networks
Focus on targeted vaccination strategies	Included to ensure answering the research questions about strategy effectiveness and design
English language	Included to ensure accurate interpretation of technical terminology
**Exclusion criteria**	
Non-network-based models	Excluded to avoid studies that do not reflect the structure and dynamics of contact networks
Non-vaccination intervention	Excluded to ensure focus on studies evaluating vaccination strategies, not other types of interventions
Purely theoretical or conceptual papers without model implementation	Excluded to ensure focus on the studies that applied modeling and reported simulation results or performance metrics
Editorials, commentaries, conference abstracts, or non-peer-reviewed work	Excluded to maintain the quality and reliability of the evidence base

### Data charting process

Based on our research questions, we developed a standardized data extraction template. The following data were extracted:

1. General information:
  Information on the publication year, authors, study aim, and study design
2. Network model features:
  Nature of network (theoretical, empirical, or hybrid), network type (scale-free, small-world, Waxman, multilayer, or real social network), and temporal nature (static or dynamic)
3. Targeted vaccination strategy:
  Topology-based (degree, betweenness, eigenvector centrality), demographic-based (age, comorbidity), behavioral/activity-based, or contact-based
4. Key metrics reported:
  Cumulative infections, peak prevalence, and epidemic duration

To ensure reliability, two independent reviewers conducted data extraction, and inter-rater consistency was verified through iterative calibration. Furthermore, we continuously reviewed our interpretation of the screening criteria, and when questions were raised, we went back to ensure the criteria had been applied consistently, regardless of who had screened the records. Conflicts and uncertainties were resolved through discussions until a consensus was reached among all researchers.

## Results

A total of 409 records were identified, of which 379 (92.7%) were found through database searches (PubMed: 77, Web of Science: 56, Scopus: 246), and 30 (7.3%) via citation tracking. A total of 215 records remained for title and abstract screening after 194 duplicates have been removed. As a result of the screening process, 124 were excluded, and 91 were evaluated in a full-text review. As a result of the eligibility assessment, 52 articles were excluded due to the following reasons: not peer-reviewed (n = 5), not focused on a vaccination-targeted intervention (n = 42), or not a network-based study (n = 5). A scoping review was conducted based on 39 studies that met the inclusion criteria, as shown in Fig 2.

### 0.1 Research Question 1: Nature, dynamics, and structural type of the networks

A network model is created to replicate a specific structure by defining nodes that represent individuals and identifying the edges that represent the interactions between individuals, where the disease may spread [31]. For practical purposes, network models can be either theoretical or empirical.

Theoretical network models are used to generate structures according to predefined rules governing node connectivity, starting from a simple model such as random networks (e.g., Erdos-Renyi model [32]), and promptly advancing to more complex models, such as the small-world network (e.g., Watts-Strogatz model) and scale-free networks (e.g., Barabasi-Albert model). Each model is rigorously analyzed and formally specified, with emphasis on their unique characteristics [33]. To achieve realistic models of social networks, researchers of agent-based models are increasingly incorporating a wide range of data, which has a significant impact on the outcome of the models. In particular, this data provides information regarding the characteristics of the agents that are collectively forming synthetic populations [34].

The network structures developed using this data are not based on observations; instead, they are created using demographic distributions, mobility data, and behavioral surveys to generate realistic synthetic populations and contact structures. The use of this approach enables scalable yet context-specific simulations of social interactions, capturing geographic and economic diversity more accurately than purely theoretical models [35].

Among the 39 included studies, the most common nature of the network was realistic synthetic populations, used in 46.1% (18/39) of studies where synthetic populations are generated and parameterized using real-world demographic and contact data. A total of 25.6% (10/39) of studies employed theoretical networks, often because of their analytical simplicity and well-defined properties.

An empirical network was used in 20.5% (8/39) of studies, which is directly constructed based on observational data sources such as GPS traces, contact diaries, or administrative datasets. However, only a small proportion of studies, 7.6% (3/39), used a mixed approach that ran simulations separately on both empirical and synthetic networks, see Table 5.

**Table 5:** Summary of study cohorts: Key characteristics of included studies (n = 39).

Author, year	Network Origin	Structure Type	Temporal Nature	Modeling approach	Vaccination Strategy	Performance measurement
Faucher et al., 2022 [44]	Realistic synthetic populations	N/A	Dynamic	Network-based SEIR	Contact-based	Attack rate, Averted cases
Klise et al., 2022 [24]	Realistic synthetic populations	N/A	Static	ABM+SEIR	Demographic-based	Cumulative deaths, Peak prevalence (hospitalization) timing
Spiliotis et al., 2022 [45]	Theoretical	Small-world	Static	ABM+SEIR	Demographic-based	Cumulative deaths
Hladish et al., 2024 [46]	Realistic synthetic populations	N/A	Dynamic	ABM+SEIR_extended	Demographic-based	Cumulative infections or deaths, Incidence averted
Feld and Hartmann, 2023 [47]	Theoretical	Small-world	Static	Network based SIR	Topology-based	Cumulative infections
Williams et al., 2025 [48]	Realistic synthetic populations	N/A	Static	ABM+SEIR_extended	Demographic-based	Averted cases
Meng et al., 2025 [25]	Theoretical	Small-world, Scale-Free	Static	ABM+SIRSV	Behavior-based	Final epidemic size
Wang et al., 2019 [49]	Realistic synthetic populations	N/A	Dynamic	ABM+SEIR	Demographic-based	Cumulative infections or deaths
Kulac¸ et al., 2025 [50]	Realistic synthetic populations	N/A	Dynamic	ABM+SEIR_extended	Demographic-based	Attack rate, Hospitalization or deaths rate
Gonzaga et al., 2024 [51]	Theoretical	Not reported	Dynamic	IABM+SEIR	Spatial-based	Averted cases or deaths, Herd immunity.
Selvaraj et al., 2022 [52]	Realistic synthetic populations	N/A	Dynamic	ABM + SIR	Spatial, demographic	Cumulative infections, Averted cases or deaths
Ajmal et al., 2024 [53]	Realistic synthetic populations	N/A	Dynamic	ABM+SEIR	Behavior-based	$R_{0}/R_{e}$
Wallrafen-Sam et al., 2025 [54]	Realistic synthetic populations	N/A	Dynamic	ABM+SEIRS	Behavior-based	Averted cases or deaths
Vilches et al., 2022 [55]	Realistic synthetic populations	N/A	Static	ABM+SEIR_extended	Demographic-based	Averted cases
Blavatska and Holovatch, 2022 [56]	Theoretical	Scale-Free	Static	SI, SIR, SIS	Topology-based	Fraction of infected (equilibrium)
Wang et al., 2021 [57]	Theoretical	Scale-Free	Static	SIR	Topology-based	Averted cases
Saunders and Schwartz, 2021 [58]	Theoretical	Scale-Free	Static	SIRS	Topology-based	Fraction of infected
Zhang et al., 2025 [59]	Realistic synthetic populations	N/A	Dynamic	ABM+ SEIRDV	Demographic, Behavior, Spatial	Cumulative infections or deaths, Peak prevalence, Epidemic duration
Tetteh et al., 2021 [60]	Theoretical	Scale-Free, Random	Static	SAIRV	Contact-based	Peak prevalence, Herd immunity
Vassallo et al., 2020 [26]	Theoretical	Erdos-Rényi, Waxman-spatial network	Static	SIR-V	Contact-based	Fraction of recovered individuals
Petrizzelli et al., 2022 [61]	Mixed	Erdos-Rényi, Random, Barabási-Albert, and SocioPatterns Dublin dataset	Dynamic for empirical	SIR	Topology-based	Number of infected, Herd immunity
Hartvigsen and Dimitroff, 2025 [62]	Theoretical	Watts-Strogatz Small-world network	Dynamic	SEIRV	Topology-based	Number of infected, Peak prevalence or timing
Fosdick et al., 2022 [63]	Realistic synthetic populations	N/A	Dynamic	SEIRextended	Demographic-based	Attack rate
Zhou et al., 2021 [64]	Empirical	Mobile phone trajectory data	Dynamic	ABM+SEIR	Hybrid (Demographic and Spatial)	Attack rate, Epidemic duration, Herd immunity
Bassett et al., 2021 [65]	Empirical	German cattle trade data	Dynamic	ABM+SEIR_extended	Demographic-based	Number of infected
Milwid et al., 2023 [66]	Realistic synthetic populations	N/A	Dynamic	ABM+SEIR	Behavior-based	Cumulative infections, Peak prevalence or timing
Yang et al., 2019 [67]	Empirical	Sociocentric social network, India	Static	ABM + SIR	Topology-based	Commulative infections, Averted cases
Bosman et al., 2024 [68]	Realistic synthetic populations	N/A	Dynamic	ABM+SEIR_extended	Demographic-based	Number of infected
Guzzetta et al., 2024 [69]	Realistic synthetic populations	N/A	Dynamic	ABM+SEIRV	Behavior-based	Cumulative infections, $R_{0}/R_{e}$
Cheng et al., 2024 [70]	Mixed	Scale‑Free networks, French high school face-to-face contact data	Dynamic for empirical	ABM + SIR	Topology-based	Number of infected individuals
Bhattacharya et al., 2024 [71]	Realistic synthetic populations	N/A	Dynamic	ABM+SEIR_extended	Behavior-based	Averted cases or deaths, Herd immunity
Hartnett et al., 2021 [19]	Empirical	Mobile device GPS data	Static	Network-based SEIR	Topology-based	Fraction of infected individuals
Sartori et al., 2022 [72]	Empirical	12 real-world networks from repositories like	Static	ABM + SIR	Topology-based	Final epidemic size
Shahzamal et al., 2020 [73]	Mixed	Momo GPS real data	Dynamic	Network-based SIR	Behavior-based	Outbreak size
Luo et al., 2018 [74]	Realistic synthetic populations	N/A	Dynamic	ABM+SEIR	Topology-based	Cumulative infections
Mones et al., 2018 [75]	Empirical	Bluetooth, plus CDR/ Facebook	Dynamic	SIR	Topology-based	Final epidemic size
Garc´ıa et al., 2022 [76]	Realistic synthetic populations	N/A	Dynamic	SEIRextended	Demographic-based	Cumulative infections
Lev and Shmueli, 2021 [77]	Empirical	Game of Thrones, Email, Facebook	Static and Dynamic	Network-based SIR	Topology-based	Cumulative infections
Laasri et al., 2024 [78]	Empirical	Socio-Patterns	Dynamic	Not reported	Topology-based	Not epidemiological performance metrics

*Note.* N/A = not applicable.

Network structure can be classified as either static or dynamic. Static networks maintain a fixed set of vertices and links that do not change over time. Many scholars studied the spread of epidemics in a relatively static state, but many real-life systems will not remain static during the spread of epidemics. Dynamic networks allow for the emergence and disappearance of both vertices and links throughout the duration of the network’s existence [36]. Such networks have been studied in the context of the spread of diseases and opinions [37,38].

In terms of temporal behavior, a total of 58.9% of studies (23/39) simulated the spread of disease on static networks, where the contact structure remains constant throughout the simulation. In contrast, 38.4% (15/39) of studies modeled dynamic contact structures, which evolved over time according to agent activity, behavior, or intervention policies, see Table 5.

### 0.2 Research Question 2: Vaccination strategy

While mass vaccination can effectively reduce and control the spread of a disease, more efficient methods exist that focus on specific individuals selected based on various factors. This approach has led to the development of targeted vaccination strategies, which prioritize immunizing those individuals who have the greatest influence on transmission networks. Compared to random vaccination, targeted vaccination can more effectively and promptly suppress disease outbreaks [23]. Based on our analysis in this study, targeted vaccination strategies are classified into the following categories:

1. Demographic-based: prioritizing target nodes according to demographic characteristics, such as age, gender, occupation, or vulnerability to disease.
2. Topology-based: prioritizing target nodes based on network metrics such as degree, centrality, and connectivity.
3. Behavior-based: prioritizing target nodes based on behaviors, actions, or eligibility criteria such as activity level or frequency of contact.
4. Spatial-based: prioritizing target nodes based on geographic zones or hotspots to ensure the protection of regions or communities that are most vulnerable to transmission risk.
5. Reactive (contact-based): prioritizing target nodes through outbreak-triggered or contact-tracing (ring vaccination).

A comprehensive review of the 39 included studies revealed that the most common vaccination strategy was topology-based, utilized in 38.4% of the studies (15/39). Additionally, demographic factors were incorporated in 33.3% of the studies (13/39). Behavior-based strategies were used in only 15.3% of the studies (6/39). Reactive or contact-based vaccinations, such as ring vaccinations triggered by case detection, were employed in 5% of the studies (2/39). Lastly, 7.6% of the studies (3 out of 39) utilized more than one vaccination strategy, indicating a mixed or hybrid approach, see Table 5.

### 0.3 Research Question 3: Performance measurements

In epidemiology, evaluation metrics serve as essential quantitative tools for understanding health trends and assessing disease prevalence. These metrics enable us to track patterns and gain a deeper understanding of health conditions, ultimately guiding public health initiatives and interventions.

Epidemic conditions are typically measured through the incidence of new cases, prevalence of existing cases, and mortality rate, which are all indicators of measuring how much disease is present in a population at a specific time [39,40].

The most important features used to assess the severity of an epidemic are its final size and timescale. The final size of the epidemic, typically measured by the number of people ultimately affected by the disease, serves as a crucial indicator of the impact of a disease on the population [41].

Epidemiological metrics for evaluating an outbreak’s peak include the peak value and peak time, which represent the highest number of cases and when it occurs, respectively. Other useful metrics include the basic reproduction number (*R*_0_), which measures infectiousness and assumes a completely susceptible population, the effective reproduction number (*R*_*e*_) is a better metric for assessing vaccination effects, since it does not assume complete susceptibility of the population, so it can be estimated for the population throughout the course of the epidemic. It is similar to (*R*_0_) but does not assume complete susceptibility [42], and the attack rate (the proportion of individuals infected among those at risk during a specific period) as a measure of disease spread within a contact network [43]. Together, they form a framework for comparing outcomes across diverse modeling studies.

Based on the explicitly reported performance measurements in the included studies, the most frequently used measurement was cumulative infections or deaths, reported in 13 studies, often alongside other metrics to quantify intervention impact. Attack rate or final epidemic size, reflecting the proportion or total number of individuals infected by the end of the outbreak and providing a direct measure of the overall spread [43], also appeared in 6 studies. Averted cases or deaths that provide a direct assessment of intervention effects in comparison with a baseline were identified in 8 studies. *R*_0_ or *R*_*e*_ appeared in 2 studies, primarily in those investigating transmissibility thresholds. A small proportion of studies reported other epidemiological metrics—such as herd immunity thresholds, hospitalization rates, and epidemic duration—that do not fall into these main categories, see Table 5.

There have been a number of studies that used non-epidemiological measurements when evaluating vaccination strategies, such as cost, but this scoping review has included only the epidemiologically relevant metrics reported in each study.

## Discussion

This scoping review identified a total of 39 modeling studies that utilized various models, including compartmental, agent-based, and network-based models, to evaluate the impact of targeted vaccination interventions on different outcomes.

By mapping findings to three research questions, the review offers a comprehensive picture of the nature of the networks represented in models (RQ1), how vaccination strategies are designed and classified (RQ2), and which epidemiological performance measurements are explicitly reported (RQ3).

According to this review, realistic synthetic populations dominate the network nature, this reflects the growing recognition of the critical need to accurately capture essential characteristics of the population, such as demographic distribution, mobility patterns, and behavioral data, to simulate realistic contact structures. This approach facilitates context-specific modeling, as the models are specifically designed to represent a particular setting, such as a designated country, city, or demographic profile [24,44,46,48]. Consequently, the results are directly suitable for that specific context. While tailored to local characteristics, they can also be adapted to represent larger populations or different scenarios, making them versatile for exploring a range of intervention strategies. This combination makes them particularly useful for policy-oriented analysis as they reflect real conditions.

Theoretical networks, such as scale-free networks, are often used in research because they can capture the structural features of contact networks and provide fundamental insights into how specific structural properties, such as degree distribution or clustering, can affect disease transmission when used as targets for intervention [26,45,47,62]. Since they are not based on actual populations, their results may lack the specificity required to create a direct impact on existing policy.

With empirical networks, which are constructed directly from observed interactions and contact data, real-world relationships and behaviors are accurately reflected without assuming network structure assumptions [19,64,65,67]. Despite this advantage, their use in the included studies was relatively limited, possibly because of the difficulty of collecting comprehensive contact data, privacy concerns, and the difficulty of scaling such networks to represent a larger population, as they sometimes focused on specific locations or population groups rather than national-scale simulations.

Topology-based and demographic-based are the most common vaccination strategies used in the included studies; they reflect methodological and practical considerations, respectively. In the earlier approach, common network metrics such as connectivity, betweenness, and centrality were used to identify individuals most critical to transmission [47,56–58,70]. As long as structural information is available, these metrics are relatively straightforward to implement in theoretical and synthetic networks. In contrast, demographic-based approaches are more practical for real-world implementation because key attributes such as age, occupation, and comorbidity are usually collected by public health authorities and can be used even when detailed contact network data are unavailable [24,45,46,48].

A behavior-based vaccination strategy may use risk-related behaviors (e.g., frequency and type of social/sexual contact), eligibility rules tied to specific activities, or adaptive uptake rates based on perceived infection risk or epidemic dynamics. Using this approach, interventions will be focused on high-risk groups whose behavior increases their likelihood of contracting or spreading infection [25,53,54].

In spatial-based vaccination strategies, targeting was often based on mobility data or urban-rural classifications, or on the detection of outbreak hotspots in the included studies [51,52], enabling the interruption of transmission chains inside concentrated geographic areas and maximizing the impact of limited vaccine supplies. Although its effectiveness depends heavily on accurate and timely surveillance data, it may be less effective when outbreaks are widespread across multiple regions.

While reactive strategies are effective in real outbreaks such as Ebola, their use in the network modeling is limited by unrealistic assumptions about detection and tracing [25,53,66].

The limited use of behavior-based, spatial-based, reactive/contact-based, and ensemble or hybrid approaches in the reviewed studies may indicate underexplored opportunities for future research.

The choice of vaccination strategy is strongly related to the performance measures used to evaluate its impact. Attack rate or final epidemic size and cumulative cases or deaths are the most frequently used metrics in the included studies, reflecting the emphasis of many studies to capture the long-term disease burden and compare the total impact of different vaccination strategies [25,44]. The peak prevalence and peak time were reported much less frequently, suggesting that short-term operational demands of outbreaks may be underrepresented in model evaluations [24,62].

A set of targeted vaccination strategies has been introduced, but their effectiveness remains unclear [79]. As part of this section, we will present comparisons of the effectiveness of various vaccination control strategies used in the included studies.

Evidence from influenza modeling highlights the benefits of age targeting. Increasing vaccination rates in school-age children by just 5%–15% would result in a reduction in 1,394,687–4,945,952 cases in the US [48]. According to another study, focusing on the vaccination of school-aged children (5–19) will reduce attack rates and deaths [49].

Together, these studies [24,46,48–50,63] demonstrate that age remains a widely used demographic indicator across contexts, ranging from prioritizing the elderly and high-risk individuals to targeting children and adolescents. However, the effectiveness of age is likely to be influenced by contact structure, behavioral uptake, and other factors.

Kifle et al. [24] found that random vaccination outperformed age-based prioritization, and similar findings show that targeting highly interactive groups, such as workers and students, can be more effective, especially when vaccine availability is moderate [50]. These findings highlight the importance of considering contact intensity and social roles in addition to age when designing demographic vaccination policies.

One of the most common topology-based strategies is targeting high-degree nodes. Simulation results indicate that this approach reduces transmission at low transmission probabilities and is among the quickest methods to control disease spread [19,57,58,61]. The effectiveness of the vaccination strategy based on the degree of the individuals was highly context-dependent, producing higher effectiveness at lower transmissibility conditions (lower *R*_0_), but less beneficial at higher transmissibility conditions [62].

Beyond degree-based targeting, other centrality measures were evaluated. PageRank and betweenness— targets bridge individuals who link different communities within the network—emerged as the most effective strategies for infection-related outcomes, minimizing both the peak and the total number of infections across networks, whereas random vaccination performed worst [72,74]. Closeness-based strategies reduce epidemic size under short-range transmission [75], while dynamic extensions of classical metrics such as infectious betweenness outperform traditional approaches [77]. These studies show that topology-based vaccination can provide greater effectiveness based on transmission conditions and network structure.

The effectiveness of spatially targeted vaccination was highlighted in [51], showing that prioritizing vulnerable populations or densely populated regions outperformed a random distribution in reducing transmission and mortality. Studies combining demographic and spatial criteria, such as space-age as a hybrid vaccination strategy, were found to be more effective at controlling outbreaks and reducing attack rates than random vaccination [64].

Considering human decisions and individuals’ risk-related behaviors within a population as criteria for designing the vaccination strategy highlights how these factors fundamentally shape epidemic outcomes. Prioritizing high-risk groups, such as MSM communities, can significantly reduce the *R*_*e*_ value [53]. Human decisions related to vaccine acceptance and hesitancy have a strong impact on decreased SARS-CoV-2 incidence, as illustrated in [54]. Models that integrate responsive decision-making show that when agents adjust their decisions in response to perceived risks, the differences between random, age-based, high-contact, and spatially focused vaccination strategies become marginal [59].

Findings suggest that behavioral context cannot be separated from vaccination policy: whether through acceptance, risk perception, or sexual/social behavior. These findings highlight the need for greater standardization and diversity in performance measurement. Incorporating a broader set of metrics—including both cumulative and peak-based measures—would allow for more comprehensive evaluations that address long-term impact, short-term effects, and strategic efficiency in parallel.

## Conclusion

This review focuses on studies that combined epidemiological models with network-based models to study and analyze the effectiveness of different targeted vaccination strategies in containing the spread of the disease. Results show that the targeted vaccination strategies outperform the random strategy; however, this effectiveness is highly dependent on the structure of the network. Findings illustrate that the demographic targeted vaccination strategy relies on the structure of the surrounding community, implying that these strategies do not perform equally across different populations.

Based on these insights, future research should focus on two key areas: (1) increasing the use of empirical networks, and (2) developing a multi-targeting approach that integrates structural factors (such as degree and bridges) with demographic information. This could lead to new insights on optimizing vaccination strategies under various epidemiological and operational constraints. Together, these steps would enhance the applicability of network-informed vaccination designs in policy and practice.

## Supporting information

S1 Checklist PRISMA Checklist. (DOCX)
